# Aerogel Assembled by Two Types of Carbon Nanoparticles for Efficient Removal of Heavy Metal Ions

**DOI:** 10.3390/gels8080459

**Published:** 2022-07-22

**Authors:** Xue-Chun Yang, Song Gao, Sha-Qi Fu, Xuan Yao, Zheng Jiao, Jing-Tai Zhao, Zhi-Jun Zhang, Ling-Li Cheng

**Affiliations:** 1 School of Environmental and Chemical Engineering, Shanghai University, Shanghai 200444, China; xuechunyang@i.shu.edu.cn (X.-C.Y.); njulegao@163.com (S.G.); fushaqi@163.com (S.-Q.F.); yx1999@shu.edu.cn (X.Y.); 2 School of Materials Science and Engineering, Guilin University of Electronic Technology, Guilin 541004, China; jtzhao@guet.edu.cn; 3 School of Materials Science and Engineering, Shanghai University, Shanghai 200444, China; zhangzhijun@shu.edu.cn

**Keywords:** self-assembled, Pb^2+^, Cu^2+^, adsorption

## Abstract

Both sodium alginate and polyethyleneimine (PEI) have a good ability to adsorb heavy metal ions. PEI and sodium alginate were used as important precursors to synthesize positively charged carbon nanoparticles (p-CNDs) with hydroxyl and carboxyl, and negatively charged carbon nanoparticles (n-CNDs) with amino, respectively. The carbon nanoparticles (CNDs) aerogel with a large specific surface area and rich functional groups were constructed by self-assembled p-CNDs and n-CNDs via electrostatic attraction for adsorption of heavy metal ions in water. The results show that CNDs aerogel has good adsorption properties for Pb^2+^ (96%), Cu^2+^ (91%), Co^2+^ (86%), Ni^2+^ (82%), and Cd^2+^ (78%). Furthermore, the fluorescence emission intensity of CNDs aerogel will gradually decrease with the increase in the adsorption rate, indicating that it can detect the adsorption process synchronously. In addition, the cytotoxicity test reveals that CNDs have good biocompatibility and will not cause secondary damage to biological cells.

## 1. Introduction

Heavy metal ions are constantly being added to the environment due to increasing urban populations and increasing industrial activity [1,2,3]. For example, urban sewage and industrial wastewater from electroplating, metallurgy, mining, and other industries are rich in a large amount of heavy metal ions [4,5]. Due to improper discharge, industrial wastewater rich in heavy metals flows into rivers, lakes, and seas, and also into the soil, which caused the content of heavy metals in fish and shrimp living in water and crops grown in soil to be beyond the standard. Furthermore, these foods will have a serious and irreversible impact on human health [6,7]. For example, Pb^2+^, Hg^2+^, Cd^2+^, etc., can cause human poisoning [8]. Some metals, such as Fe^3+^, Cu^2+^, Zn^2+^, and Cr^3+^, are essential for optimal growth, development, and reproduction. However, when the concentration exceeds the water standard, they will become harmful to health [9]. Therefore, it is very important to study an efficient method to remove heavy metal ions.

Currently, there are many approaches for removing heavy metal ions from wastewater, including chemical precipitation, ion exchange, filtration, biological treatment, and adsorption [10,11,12,13,14]. Among them, the adsorption technology is considered to be a promising technique for removing heavy metal ions from wastewater due to its low cost, ease of use, and high efficiency [15,16]. In addition, increasingly more efficient adsorbent materials have also been studied and reported. For example, organic polymer adsorbents (chitosan, alginate, agarose, polyethyleneimine (PEI), etc.), inorganic porous materials (activated carbon, zeolites, molecular sieves, etc.) and organic-inorganic hybrid gel adsorbents (graphene-based aerogels or hydrogels, etc.) [2,3,4,5,6,7]. Among them, carbon nanoparticles (CNDs), as a rookie that have attracted widespread attention in recent years, have a good application prospect in the removal of heavy metal ions due to their good hydrophilicity, large specific surface area, and rich functional groups [17,18,19,20,21,22]. The excellent adsorption performance for heavy metal ions of CNDs was proved via both Langmuir and Freundlich isotherms due to their good hydrophilicity, large specific surface area, and abundant functional groups [23]. For example, Song et al. prepared the carbon quantum dots/nanofibrillated cellulose composite (CQDs/NFC) aerogel to adsorb the Cr^3+^ at different pH values. More importantly, the CQDs can detect the Cr^3+^ adsorption behavior of aerogel [24]. Wang, L. et al. coated mesoporous organosilica with 1–2 layers of CNDs to remove heavy metal ions through the electrostatic force and complex formation between metal ions and amide groups. The adsorption sequence is Hg (II) (56%) > Cu (II) (53%) > Pb (II) (43%) [25]. Furthermore, we also reported the detection and removal of heavy metal ions by pure CNDs. The response of CNDs to Pb^2+^ is best with a detection limit of 3 ppb and a removal rate of 94.8% [26]. Although many reports are regarding the combination of CNDs and other adsorbents, the adsorption properties of pure CNDs have rarely been studied.

Herein, the adsorption properties of the aerogel prepared by pure CNDs have been fully studied. The negatively charged carbon nanoparticles (n-CNDs) carrying amino functional groups were successfully synthesized by one-step sintering. The positively charged carbon nanoparticles (p-CNDs) carrying amino functional groups were simply prepared by a secondary hydrothermal method. The n-CNDs and p-CNDs formed fluorescent yellow CNDs aerogel with super large specific surface areas by electrostatic self-assembly. The CNDs aerogel has a strong adsorption performance for heavy metal ions, especially Pb^2+^ with an adsorption rate of 96%. In addition, the fluorescence emission intensity of CNDs aerogel shows a linear downward trend with the increase in the adsorption rate, which can well monitor the adsorption behavior of CNDs aerogel on heavy metal ions.

## 2. Results and Discussion

**Figure 1:**
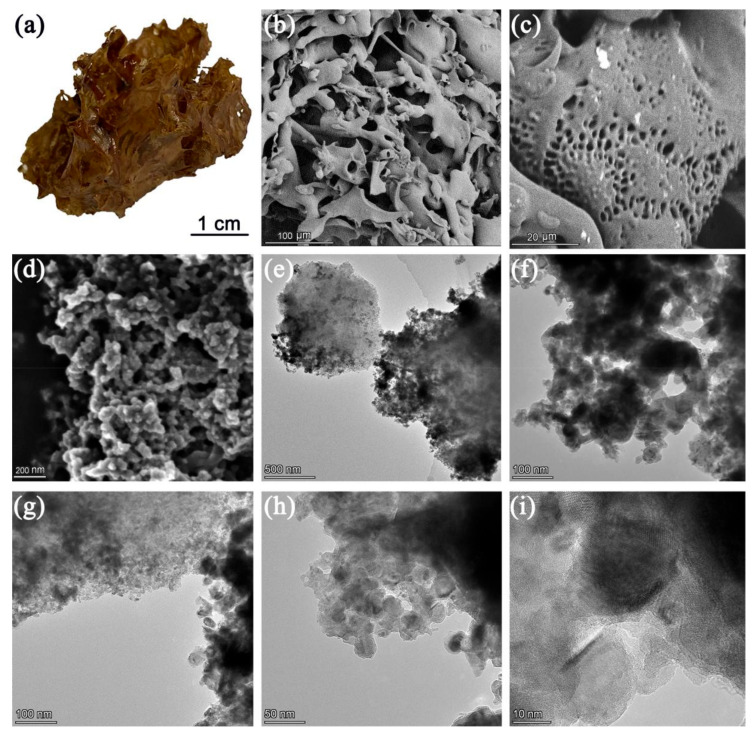
(**a**) The photographs of CNDs aerogel, (**b**–**d**) the SEM images of CNDs aerogel, and (**e**–**i**) the TEM images of CNDs aerogel.

Figure 1 shows a bulk of CNDs aerogel constructed by the electrostatic attraction self-assembly of p-CNDs and n-CNDs. It is observed from the SEM images that the CNDs aerogel with a 3D structure and abundant pores are constructed by the curly sheets structure (Figure 1b). After further magnification, we found that the porous curly sheets were formed by the cross-linking of nanospheres (Figure 1c,d). The fine structure of CNDs aerogel also can be clearly observed from the TEM images. Figure 1e–g reveal that CNDs aerogel have a rich pore structure. Moreover, the smallest structural unit of CNDs aerogel are CNDs (n-CNDs or p-CNDs) (Figure 1h,i). The BET test was used to explore the specific surface area and pore distribution of the CNDs aerogel (Figure S1). The pores of CNDs aerogel are mostly distributed between 5–10 nm, and its specific surface area is 138 m^2^/g. The abundant pore structure and super large specific surface area enables the CNDs aerogel to fully contact and soak in heavy metal ion solution, and also provide abundant active sites for the adsorption of heavy metal ions, which is conducive to the adsorption process.

**Figure 2:**
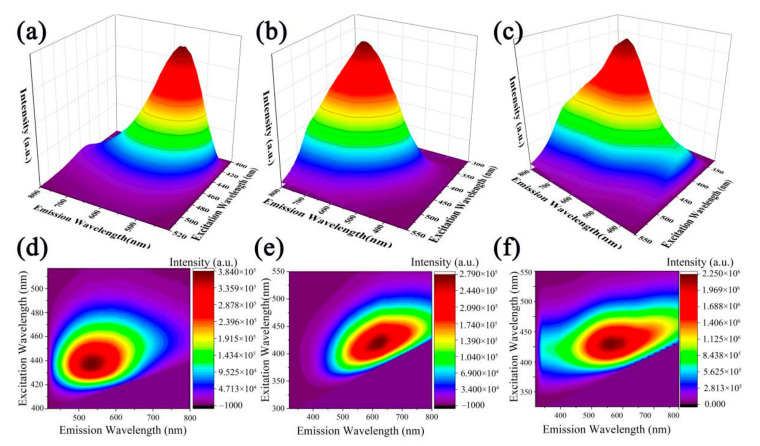
The fluorescence emission-excitation 3D map of (**a**) p-CNDs, (**b**) n-CNDs, (**c**) CNDs aerogel. The 2D contour map of the fluorescence emission spectrum of (**d**) p-CNDs, (**e**) n-CNDs, (**f**) CNDs aerogel.

We tested the fluorescence emission-excitation 3D map and 2D contour map of the fluorescence emission spectra of p-CNDs, n-CNDs, and CNDs aerogel to test their fluorescent properties. In Figure 2a,d, the emission center of p-CNDs is concentrated in the green region of 500–550 nm, and the emission range is 420–750 nm. Similarly, the n-CNDs emit bright yellow-green light between 530–620 nm with a wavelength range of 400–800 nm. Additionally, the CNDs aerogel emits light-yellow fluorescence with a wavelength range of 440 nm–800 nm. 

**Figure 3:**
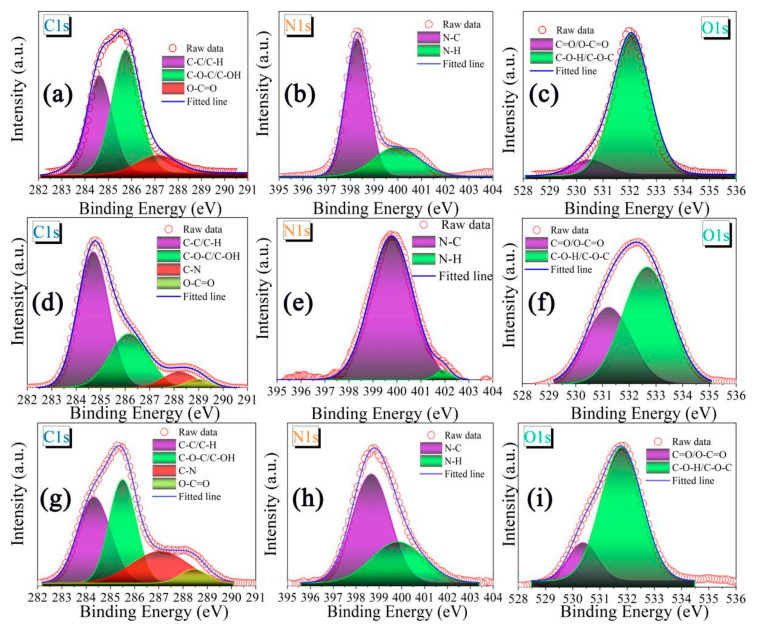
The high-resolution C1s, N1s, and O1s XPS spectra of (**a**–**c**) p-CNDs, (**d**–**f**) n-CNDs, and (**g**–**i**) CNDs aerogel.

In order to study the chemical structure of p-CNDs, n-CNDs, and CNDs aerogel carefully, the XPS and FTIR test were carried out, respectively. It can be found that the p-CNDs mainly contain C=O (287.1 eV) and C–O–C/C–OH (285.8 eV) (Figure 3a), N–C (398.3 eV) and N–H (400 eV) (Figure 3b) [27,28]. Furthermore, the XPS high resolution spectrum of O1s also proves the existence of C=O (530.5 eV) and C–O–C/C–OH (532 eV) (Figure 3c) [28,29]. In contract, the proportion of carbon containing functional groups of n-CNDs is higher (Figure 3d), which can be also proved by the XPS survey (Figure S3a,b). As shown in Figure 3f, n-CNDs carry a large amount of C=O (531.1 eV) and C–O (532.8 eV), which is also the reason n-CNDs display as negatively charged (Figure S2). The proportion of functional groups on the surface of CNDs aerogel constructed by electrostatic self-assembly of p-CNDs and n-CNDs changed significantly, whereas the types of functional groups remained the same. It is indicated that the p-CNDs and n-CNDs just cross-linked each other through electrostatic attraction and hydrogen bonding, and their chemical structures are not destroyed (Figure 3g–i). The similar conclusions can be further verified by FTIR spectra of p-CNDs, n-CNDs, and CNDs aerogel (Figure S4). The main functional groups of n-CNDs are C–OH/O–H (3360 cm−^1^), O–C=O (1400 and 1630 cm^−1^), and C–O–C (1010 and 1124 cm^−1^). Besides the above three functional groups, p-CNDs also have the amino group. Moreover, the amino group in CNDs aerogel becomes increasingly stronger with the increase in p-CNDs content. There are no new functional groups that appeared in CNDs aerogel, and the functional groups from n-CNDs and p-CNDs were not destroyed. 

**Figure 4:**
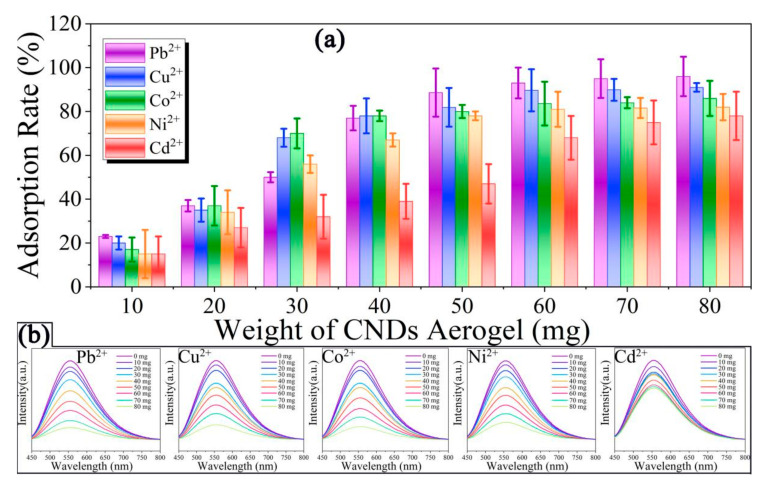
(**a**) The adsorption rate of CNDs aerogel for Pb^2+^, Cu^2+^, Co^2+^, Ni^2+^, Cd^2+^ with the addition of CNDs aerogel. (**b**) The change of fluorescence intensity of CNDs aerogel with the content of Pb^2+^, Cu^2+^, Co^2+^, Ni^2+^, Cd^2+^.

The CNDs aerogel is endowed with excellent adsorption ability by a large number of functional groups such as amino, carboxyl, hydroxyl, etc. As shown in Figure 4a, the adsorption rate of CNDs aerogel for heavy metal ions gradually increases with the increase in CNDs aerogel addition. The adsorption saturation was almost reached when 80 mg of CNDs aerogel was added to the heavy metal ions solution (0.015 mg/mL). At this time, the adsorption rates of CNDs aerogel for five heavy metal ions are as follows: Pb^2+^ (96%) > Cu^2+^ (91%) > Co^2+^ (86%) > Ni^2+^ (82%) > Cd^2+^ (78%). We studied the adsorption rates of CNDs aerogel in aqueous solutions of heavy metal ions with different pH solutions (Figure S6). The adsorption rates of CNDs aerogel changes significantly in different pH solutions. The reason for this is that the pH value can obviously affect the degree of protonation and the surface potential of CNDs aerogel functional groups. In addition, the existing forms of heavy metal ions and the content of hydrogen ions are also changed with the change of pH. The fluorescence emission intensity of CNDs aerogel decreases gradually with the increase in the adsorption rate (Figure 4b). Among them, CNDs aerogel has the highest adsorption rate for Pb^2+^. Correspondingly, the decrease in the fluorescence emission intensity of CNDs aerogel that adsorbed Pb^2+^ is the most obvious. Followed by Cu^2+^, Co^2+^, Ni^2+^, and Cd^2+^. The significant change of fluorescence emission intensity of CNDs aerogel can be used as an effective method to detect the adsorption process.

**Figure 5:**
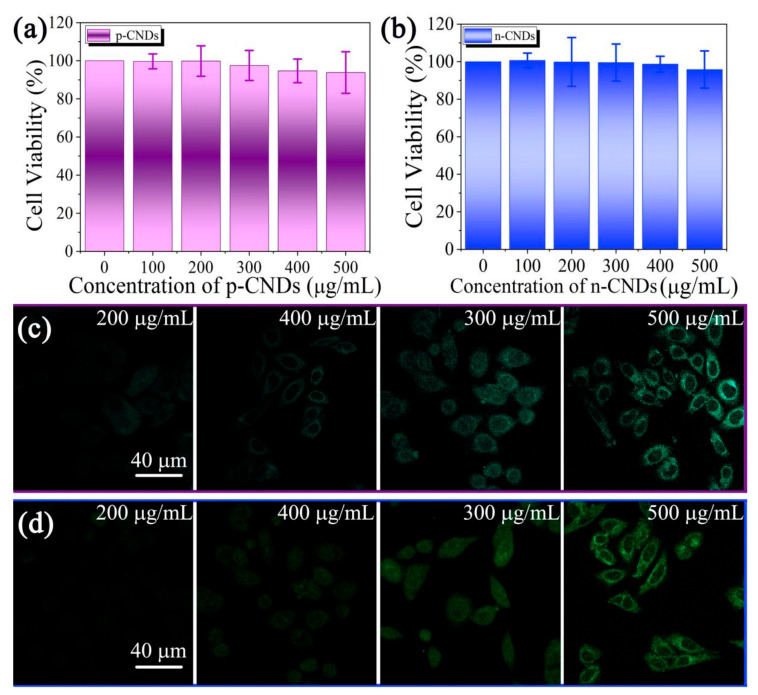
Cytotoxicity of (**a**) p-CNDs and (**b**) n-CNDs on HeLa cells after incubation for 24 h. Control group: HeLa cells without CNDs. (**c**) Confocal fluorescence images of HeLa cells after culturing with 200 μg mL^−1^, 300 μg mL^−1^, 400 μg mL^−1^, 500 μg mL^−1^ of (**c**) p-CNDs and (**d**) n-CNDs for 4 h, respectively.

In the process of adsorption to heavy metal ions, a little amount of CNDs will inevitably fall off from the CNDs aerogel and disperse into the water. In order to research whether the residual CNDs will cause secondary pollution to the environment, cytotoxicity experiments were carried out. Specifically, HeLa cells were incubated with different concentrations of p-CNDs and n-CNDs for 24 h (as shown in Figure 5a,b). The results clearly show that the cell viability did not decrease significantly for HeLa cells treated with CNDs. Especially, the cell viability is more than 94% even at high CNDs concentrations of 500 μg mL^−1^, which proves that both p-CNDs and n-CNDs have good biocompatibility, and the CNDs aerogel is an environmentally friendly and non-toxic adsorbent for heavy metal ions [30]. The further evidences were observed in cell imaging results (Figure 5c,d). As shown in Figure 5c, the HeLa cells cultured with 200 μg mL^−1^, 300 μg mL^−1^, 400 μg mL^−1^, and 500 μg mL^−1^ p-CNDs for 4 h can emit bright blue-green light under a 430 nm laser. With the increase in the p-CNDs addition, the fluorescence intensity of the HeLa cells also increased significantly. Moreover, the p-CNDs are distributed in the cytoplasm and do not enter the nucleus, which also indicates that p-CNDs have no cytotoxicity [31,32]. A similar phenomenon can also be observed in Figure 5d.

## 3. Conclusions

In short, the p-CNDs were prepared successfully with PEI as an important passivator by the secondary hydrothermal method. The n-CNDs were synthesized by simple thermal sintering using sodium alginate as an important precursor. The CNDs aerogel with a large specific surface area (138 m^2^/g) and rich pore structure were constructed through an electrostatic self-assembly between p-CNDs and n-CNDs. The adsorption rates of CNDs aerogel for Pb^2+^, Cu^2+^, Co^2+^, Ni^2+^, and Cd^2+^ were all over 78%. Among them, the adsorption performance of CNDs aerogel for Pb^2+^ was the best, which reached up to 96%. The fluorescence emission intensity of CNDs aerogel decreased significantly with the increase in the adsorption rate, which can be used as an important means to detect the adsorption process. In addition, the p-CNDs and n-CNDs both have good biocompatibility and will not cause secondary pollution. This simple and creative strategy provides a new idea for the design and preparation of new aerogel and expands the application of CNDs in all solid states. However, the pure CNDs aerogel is not strong enough, and it will perform better when placed among the other remediation tools [25].

## 4. Materials and Methods

### 4.1. Materials

Ethanol, sodium alginate, urea, glucose, polyethyleneimine (PEI), Pb(NO_3_)_2_ (99.99%), CuCl_2_·2H_2_O (AR grade), Ni(NO_3_)_2_·6H_2_O (AR grade), CoSO_4_·7H_2_O (AR grade), and CdCl_2_·2.5H_2_O (98%) were purchased from Aladdin Chemistry Co., Ltd. (Los Angels, CA, USA). Deionized water was used during the experiment.

### 4.2. Methods

#### 4.2.1. Synthesis of p-CNDs and n-CNDs

The p-CNDs were obtained by a two-step hydrothermal method: first, 0.7 g glucose was added into the mixed solution of water and ethanol (volume ratio 1:1) and heated at 180 °C for 3 h. Then, 0.4 g PEI was added into the above solution and heated at 100 °C for 3 h. After cooling to the room temperature, the p-CNDs solution was obtained. 

The n-CNDs were prepared by a one-step sintering method with sodium alginate and urea as precursors, and the sintering temperature was 220 °C.

#### 4.2.2. Synthesis of CNDs Aerogel

The p-CNDs aqueous solution (0.03 g/mL) and n-CNDs aqueous solution (0.03 g/mL) are mixed and freeze-dried to obtain CNDs aerogel. A series of CNDs aerogels were prepared by varying the weight ratio of p-CNDs to n-CNDs (W_p-CNDs_: W_n-CNDs_ = 0.8, 1, and 1.25), which are denoted as 1-CNDs aerogel, 2-CNDs aerogel, and 3-CNDs aerogel, respectively. Because 2-CNDs aerogel has the best adsorption performance, it is taken as the research object for the characterization tests in our paper (Figure S5).

#### 4.2.3. Cytotoxicity Test

The method of the cytotoxicity test is the same as our previous work [21].

## Data Availability

The data presented in this study are available on request from the corresponding author.

## Supplementary Materials

The following supporting information can be downloaded at: https://www.mdpi.com/article/10.3390/gels8080459/s1, Figure S1: The adsorption and desorption curve and (b) pore size distribution curve of CNDs aerogel; Figure S2: Z-potential of p-CNDs, n-CNDs and CNDs aerogel; Figure S3: The survey XPS spectrum of (a) p-CNDs, (b) n-CNDs and (c) CNDs aerogel; Figure S4: The FTIR spectra of p-CNDs, n-CNDs and CNDs aerogel; Figure S5: The absorption rate of CNDs aerogel with different composite ratios for heavy metal ions.
