# Supplementary material for: Aerogel Assembled by Two Types of Carbon Nanoparticles for Efficient Removal of Heavy Metal Ions
Source: Gels. 2022 Jul 22;8(8):459. doi: 10.3390/gels8080459 (PMC9329938; doi:10.3390/gels8080459)
Supplement: Supplementary file 1 [file gels-08-00459-s001.zip › gels-1806654-supplementary.pdf]

Supplementary Materials

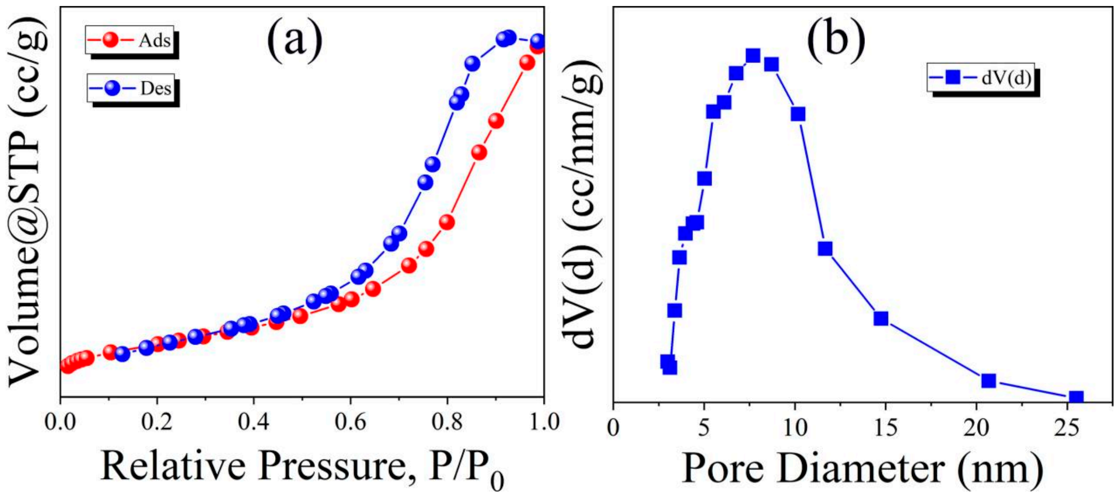

Figure S1. The adsorption and desorption curve and (b) pore size distribution curve of CNDs aerogel.

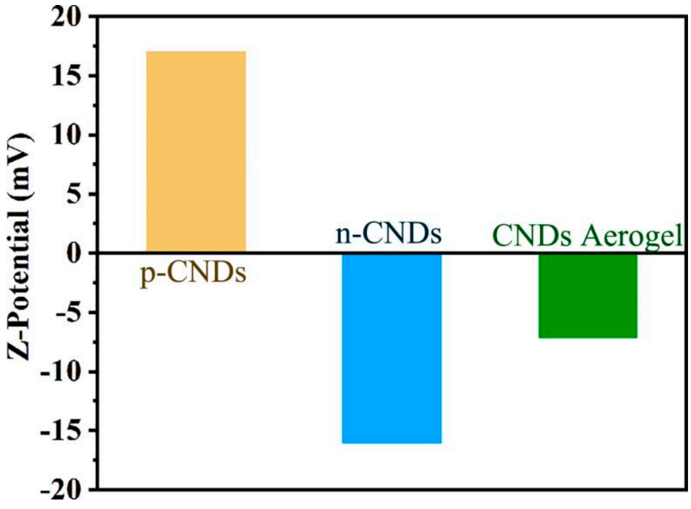

Figure S2. Z-potential of p-CNDs, n-CNDs and CNDs aerogel at pH=7.

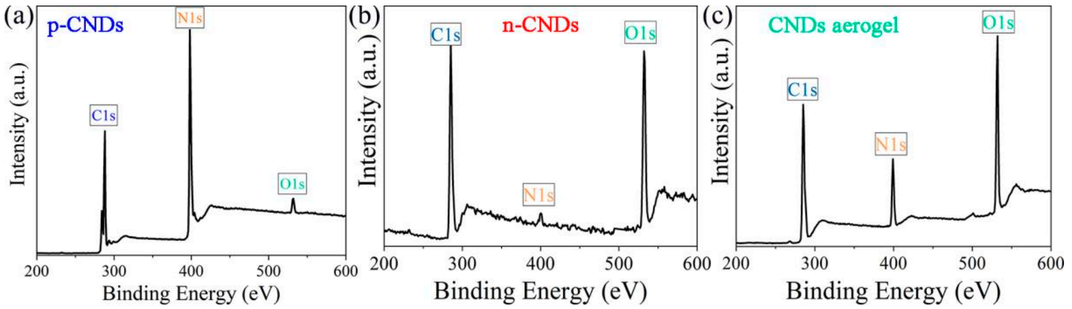

Figure S3. The survey XPS spectrum of (a) p-CNDs, (b) n-CNDs and (c) CNDs aerogel.

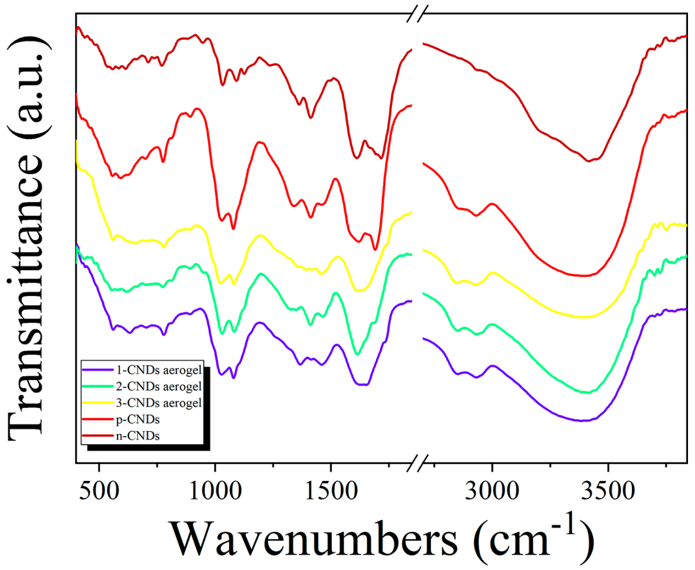

Figure S4. The FTIR spectra of p-CNDs, n-CNDs and CNDs aerogel.

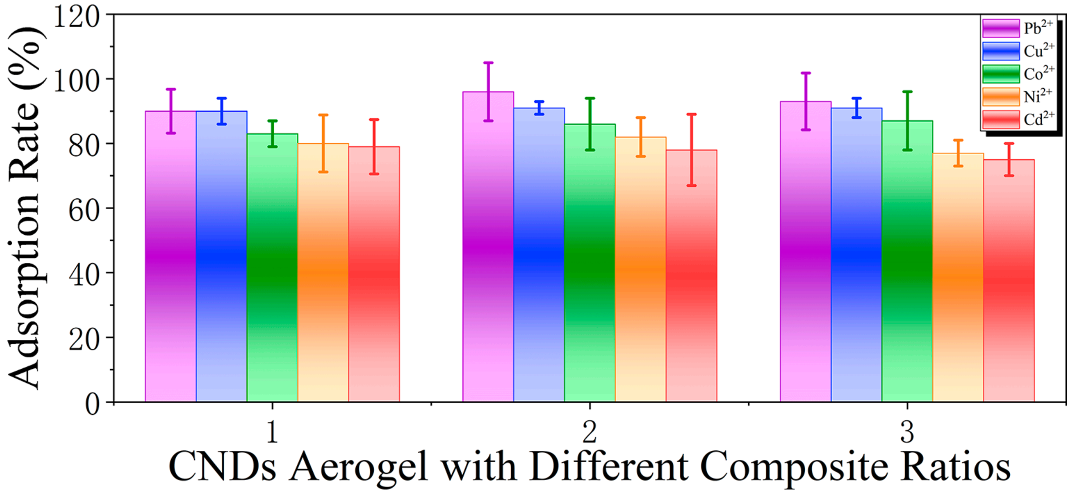

Figure S5. The adsorption rate of CNDs aerogel with different composite ratios for heavy metal ions.

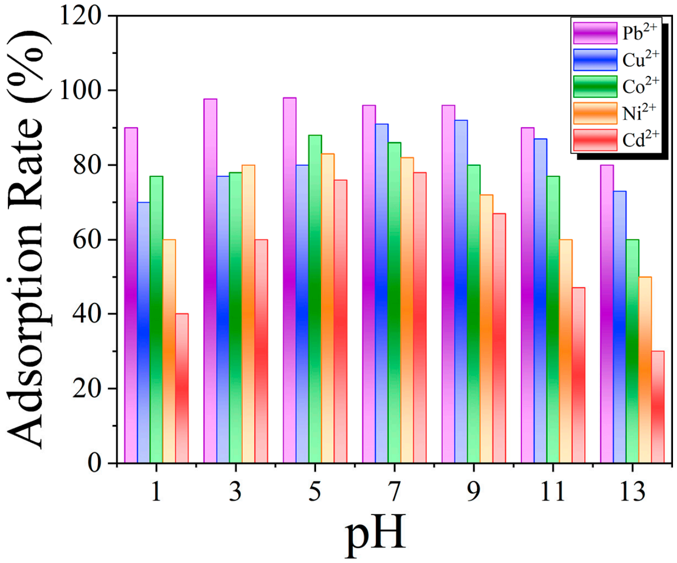

Figure S6. The adsorption rate of CNDs aerogel at different pH.

Structural characterizations

A transmission electron microscope (TEM) (FEI, TF20, United States) and a field emission scanning electron microscope (Zeiss Gemini SEM 300) were employed to observe morphologies of p-CNDs, n-CNDs and CNDs aerogel. Fourier transform infrared (FT-IR) spectra were recorded on a Nicolet Nexus 670 FTIR spectrometer. X-ray photoelectron spectroscopy (XPS) was performed using a Kα spectrometer (Thermo Scientific K-Alpha). The photo-luminescence (PL) spectra were obtained on a FLS1000 fluorescence spectrometer. An XRF spectrometer (Bruker Tiger S8) was used to measure and calibrate the content of all heavy metals in the solution.
